# The membrane targeted apoptosis modulators erucylphosphocholine and erucylphosphohomocholine increase the radiation response of human glioblastoma cell lines *in vitro*

**DOI:** 10.1186/1748-717X-1-6

**Published:** 2006-03-29

**Authors:** Amelie Rübel, René Handrick, Lars H Lindner, Matthias Steiger, Hansjörg Eibl, Wilfried Budach, Claus Belka, Verena Jendrossek

**Affiliations:** 1 Department of Radiation Oncology, Experimental Radiation Oncology, University of Tuebingen, Hoppe-Seyler-Str. 3, D-72076 Tuebingen, Germany; 2 Department of Internal Medicine III, University Hospital Grosshadern, Marchioninistraße 15, D-81377 Munich, Germany; 3 Max-Planck-Institute for Biophysical Chemistry, Am Fassberg 11, D-37077 Goettingen, Germany; 4 Department of Radiation Oncology, Moorenstrasse 5, D-40225 Duesseldorf, Germany

## Abstract

**Background:**

Alkylphosphocholines constitute a novel class of antineoplastic synthetic phospholipid derivatives that induce apoptosis of human tumor cell lines by targeting cellular membranes. We could recently show that the first intravenously applicable alkylphosphocholine erucylphosphocholine (ErPC) is a potent inducer of apoptosis in highly resistant human astrocytoma/glioblastoma cell lines *in vitro*. ErPC was shown to cross the blood brain barrier upon repeated intravenous injections in rats and thus constitutes a promising candidate for glioblastoma therapy. Aim of the present study was to analyze putative beneficial effects of ErPC and its clinically more advanced derivative erucylphosphohomocholine (erucyl-N, N, N-trimethylpropanolaminphosphate, ErPC3, Erufosine™ on radiation-induced apoptosis and eradication of clonogenic tumor cells in human astrocytoma/glioblastoma cell lines *in vitro*.

**Results:**

While all cell lines showed high intrinsic resistance against radiation-induced apoptosis as determined by fluorescence microscopy, treatment with ErPC and ErPC3 strongly increased sensitivity of the cells to radiation-induced cell death (apoptosis and necrosis). T98G cells were most responsive to the combined treatment revealing highly synergistic effects while A172 showed mostly additive to synergistic effects, and U87MG cells sub-additive, additive or synergistic effects, depending on the respective radiation-dose, drug-concentration and treatment time. Combined treatment enhanced therapy-induced damage of the mitochondria and caspase-activation. Importantly, combined treatment also increased radiation-induced eradication of clonogenic T98G cells as determined by standard colony formation assays.

**Conclusion:**

Our observations make the combined treatment with ionizing radiation and the membrane targeted apoptosis modulators ErPC and ErPC3 a promising approach for the treatment of patients suffering from malignant glioma. The use of this innovative treatment concept in an *in vivo *xenograft setting is under current investigation.

## Background

During the last decades there has been only little progress in the therapy of malignant glioma including the most aggressive manifestation glioblastoma multiforme (GBM). This infiltrative and destructive growing tumor is still almost uniformly fatal with a life expectancy of a few weeks to several months. Standard therapy consisting of surgery with postoperative external-beam radiation therapy (RT) prolongs median survival times to 9–12 months with almost no benefit of refined surgery, aggressive chemotherapy or improved technology of radiation therapy [1-4]. In this regard, low intrinsic sensitivity of the malignant cells to ionizing radiation and standard DNA-damaging drugs constitutes one of the critical parameters for treatment failure. Thus, novel treatment approaches are badly needed to improve prognosis of GBM patients. Since defective apoptosis can contribute to treatment resistance aberrant apoptosis signaling pathways of tumor cells constitute an attractive target for the modulation of therapy response.

There is accumulated evidence that treatment with ionizing radiation or DNA-damaging drugs triggers activation of the intrinsic, death receptor-independent death pathway. This pathway critically involves alterations of mitochondrial function including breakdown of the mitochondrial membrane potential and release of cytochrome c. A cytoplasmic complex composed of cytochrome c, the adapter protein Apaf-1, dATP and pro-caspase-9, the apoptosome, enables the proteolytic activation of initiator caspase-9 that subsequently triggers the effector caspase cascade [5]. Pro- and anti-apoptotic proteins of the Bcl-2 family function as important regulators of this mitochondrial death pathway.

The major signaling pathway triggering DNA-damage-induced apoptosis upstream of the mitochondria involves transcriptional activation of the tumor suppressor p53. P53 triggers up-regulated expression of the pro-apoptotic Bcl-2 family member Bax and Bax-induced mitochondrial damage [6-8]. Apart from Bax, further p53-regulated pro-apoptotic Bcl-2 proteins such as the BH-3 only proteins Puma and Noxa can similarly participate in the regulation of mitochondrial permeability and trigger the intrinsic, mitochondrial death pathway for apoptosis execution [9-11]. In addition to transcriptional activation of p53, release of the proapoptotic lipid second messenger ceramide from cellular membranes via the action of acid sphingomyelinase (ASM) has been described as an important mediator of radiation-induced apoptosis upstream of the mitochondria (for review see [12]) involving Bax-mediated mitochondrial alterations [13].

During tumorigenesis tumor cells often acquire mutations related to apoptosis resistance. Among the signaling molecules found to be altered or defective in malignant glioma, members of the apoptosis signaling cascade (p53, Bcl-2; for review see [14]) as well as survival modulators indirectly involved in apoptosis regulation (PI3K/PKB-pathway; for review see [15]) have been identified [16-18]. Consequently, novel anti-neoplastic agents that target those aberrant apoptosis and/or survival pathways may be suited to overcome intrinsic resistance of malignant glioma. In particular, a combination of radiation therapy with an apoptosis modulator that overrides radiation resistance should be useful to increase the therapeutic response to ionizing radiation [19].

In this regard, alkylphosphocholines (APC), a structural class of antineoplastic synthetic phospholipid analogs, have been identified as promising apoptosis modulators with a high potential value for the treatment of malignant glioma. These membrane targeted drugs exert potent cytostatic and cytotoxic effects *in vitro *as well as in animal models. They affect both apoptotic and survival signal transduction pathways, including activation of the pro-apoptotic SAPK/JNK pathway and inhibition of the mitogenic MAPK/ERK and PI3K-Akt/PKB survival pathways (for a review see [20,21]).

Interestingly, synthetic phospholipid analogs display almost no cross resistance towards standard DNA-damaging drugs and ionizing radiation *in vitro *[22-26] and unpublished data). In contrast, combined treatment with DNA-damaging anticancer drugs and ionizing radiation point to additive or synergistic effects [22,25,27,28]. These promising *in vitro *and preclinical data suggest that these membrane targeted apoptosis modulators may be suited for administration as single drugs as well as in combination with radiation therapy to overcome resistance to standard treatment concepts.

Since in the case of malignant glioma, the use of apoptosis targeting agents that cross the blood-brain barrier is mandatory, the prototypical intravenously applicable APC-derivative ErPC is most promising for the treatment of malignant glioma: Apart from potent cytotoxic efficacy on human malignant astrocytoma/glioblastoma cell lines *in vitro *[20,24,29,30] pharmacokinetic experiments with healthy rats revealed that ErPC is able to cross the blood brain barrier. Upon repeated intravenous applications of nontoxic drug doses an accumulation in brain tissue could be observed. Moreover, in glioma-bearing rats an accumulation in tumor tissue was also demonstrated [31,32].

To provide a scientific basis for the use of ErPC and its structural derivative ErPC3 in combination with ionizing radiation, aim of the present study was to analyze putative beneficial effects of ErPC and ErPC3 on radiation induced apoptosis and eradication of clonogenic tumor cells in human astrocytoma/glioblastoma cell lines *in vitro*.

## Results

### ErPC induces time- and concentration-dependent apoptosis in human malignant glioma cell lines

We have shown earlier that induction of apoptosis via the intrinsic pathway contributes to the antineoplastic activity of ErPC [24,29,33]. The present study was designed to substantiate our findings on the importance of apoptosis for cytotoxic efficacy of ErPC in human malignant glioma. To this end, time course and dose response relationships for ErPC-induced cell death were analyzed in three astrocytoma/glioblastoma (AC/GBM) cell lines (A172, T98G and U87MG) by fluorescence microscopy. Combined staining with Hoechst33342 and PI allowed to differentiate between apoptosis and necrosis.

**Figure 1:**
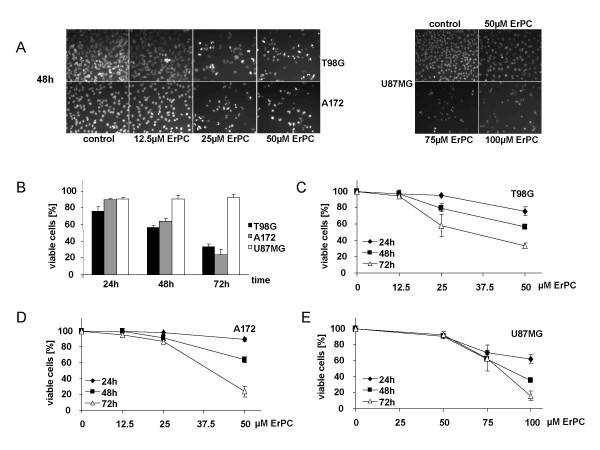
**ErPC induces growth arrest and apoptosis in human malignant glioma cell lines**. T98G, A172 and U87MG were treated with 0, 12.5, 25, 50, 75 or 100 μM ErPC for 24 h, 48 h and 72 h as indicated. Subsequently, induction of apoptosis and necrosis was analyzed by fluorescence microscopy upon combined staining with Hoechst33342 and propidium iodide (PI). Apoptotic and necrotic cell death was quantified by counting cells with apoptotic and necrotic morphology. The percentage of viable cells was calculated from the difference of total cell count (= 100%) and apoptotic (% apoptosis) plus necrotic cells (% necrosis) (% viable cells = 100% – (% apoptosis + % necrosis). While 25 to 50 μM ErPC were sufficient to induce growth arrest and apoptosis in T98G and A172 cells, 75 to 100 μM ErPC were required to induce similar effects in U87MG cells. Data show one representative of three independent experiments **(A) **or means ± s.d., n = 3 **(B, C, D, E)**. **(A) **Morphologic appearance of human malignant glioma cell lines 48 h after treatment with the indicated ErPC-concentrations. **(B) **Time-dependent decrease in the amount of viable cells upon treatment with 50 μM ErPC. **(C, D, E) **Concentration-dependent decrease in the amount of viable **(C) **T98G **(D) **A172 and **(E) **U87MG cells upon ErPC-treatment.

Consistent with our earlier findings concentrations of 25 to 50 μM ErPC were sufficient to induce growth arrest and apoptosis in A172 and T98G cells within 48 h of treatment. This is visualized in Fig. 1A by decreased cell density and increased numbers of cells with condensed chromatin and nuclear fragmentation indicative for apoptosis upon treatment with increasing ErPC-concentrations. In contrast, 75 to 100 μM ErPC were required to induce similar effects in U87MG cells (Fig 1A). Concordantly, 50 μM ErPC strongly decreased the number of viable A172 and T98G cells with most pronounced effects at extended incubation times (72 h) (Fig. 1B). In contrast, U87MG cells remained mainly unaffected by treatment with 50 μM ErPC even after 72 h of treatment (Fig. 1B). In general, all AC/GBM cell lines tested were sensitive to the cytotoxic effects of ErPC. ErPC triggered time- and concentration-dependent cell death in all cell lines with T98G and A172 cells being more sensitive than U87MG cells at all time points (Fig 1C–E).

### Human malignant glioma cell lines are resistant to radiation-induced apoptosis

**Figure 2:**
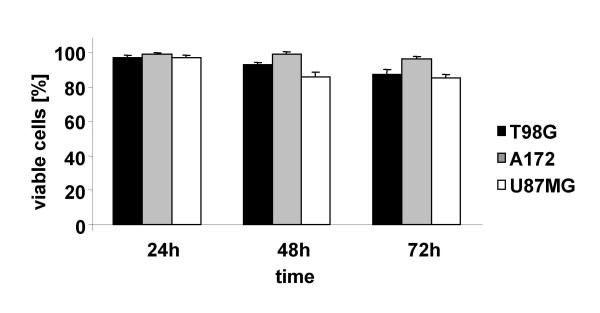
**Human malignant glioma cell lines are resistant to radiation-induced cell death**. T98G, A172 and U87MG were irradiated with 10 Gy. 24 h, 48 h and 72 h after treatment, induction of apoptosis and necrosis was quantified by fluorescence microscopy counting the cells with apoptotic and necrotic appearance upon combined staining with Hoechst33342 and PI. The percentage of viable cells was calculated as indicated in Fig.1. Data represent means ± s.d., n = 3.

Intrinsic resistance of malignant glioma cells to ionizing radiation contributes to treatment failure. To establish time course and dose response relationships for radiation-induced cell death in human malignant glioma cell lines used in the present study, apoptotic and necrotic cell death was quantified 24, 48 and 72 h after single dose application of 2.5, 5 or 10 Gy. In contrast to treatment with ErPC, T98G, A172 and U87MG cells turned out to be rather resistant against radiation-induced apoptosis and necrosis (Fig. 2). Even 72 h after a single dose of 10 Gy, irradiation almost completely failed to trigger cell death in T98G cells, A172 cells and U87MG cells resulting in cell death rates below 20%.

### ErPC sensitizes human malignant glioma cell lines to radiation-induced apoptosis

It has been shown that ionizing radiation as well as the membrane targeted apoptosis modulator ErPC induce apoptosis via the intrinsic, mitochondrial death pathway. Despite these similarities in apoptosis execution, ErPC was able to induce apoptosis and necrosis in malignant glioma cell lines resistant to radiation-induced cell death (Fig. 1). This observation constituted the rationale to evaluate whether combined treatment with ErPC could increase radiation-induced cell death in human malignant glioma cell lines. To this end, T98G, A172 and U87MG cells were treated with 2.5, 5 and 10 Gy and/or 0, 12.5, 25, 50, 75 or 100 μM ErPC. ErPC was added to the culture medium 10 min after irradiation and induction of apoptosis and necrosis was determined 24 h, 48 h and 72 h after treatment.

**Figure 3:**
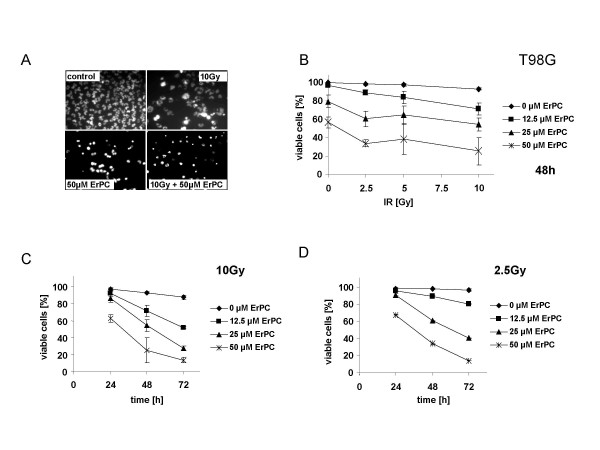
**ErPC and radiation cooperate to induce cell death in T98G cells**. T98G were irradiated with a single dose of 0, 2.5, 5 or 10 Gy and subsequently treated with 0, 12.5, 25 or 50 μM ErPC as indicated. Induction of apoptosis and necrosis was quantified 24 h, 48 h and 72 h after treatment by fluorescence microscopy counting the cells with apoptotic and necrotic morphology upon combined staining with Hoechst 33342 and PI. The percentage of viable cells was calculated as indicated in Fig.1. Data show **(A) **one representative of three independent experiments or **(B, C, D) **means ± s.d. ; n = 3. **(A) **Photomicrographs of morphologic appearance of T98G cells upon treatment with medium (control), 10 Gy, 50 μM ErPC or 10 Gy and 50 μM ErPC. **(B) **Dose dependent increase in efficacy of the combination 48 h after treatment. **(C **and **D) **Time dependent increase in efficacy of the combination.

As shown in Fig. 3A combined treatment of T98G cells for 48 h with 10 Gy and 50 μM ErPC clearly increased the levels of radiation-induced apoptosis. Quantitative analysis indicated that enhanced cell death induction 48 h after combined treatment compared to either treatment alone occurred in a dose- and concentration-dependent manner yielding maximum levels of apoptosis in the presence of 50 μM ErPC (Fig. 3B). Moreover, at all radiation doses tested efficacy of combined treatment depended on the ErPC-concentration and treatment time with most pronounced effects at 72 h (Fig. 3C+D and data not shown).

**Figure 4:**
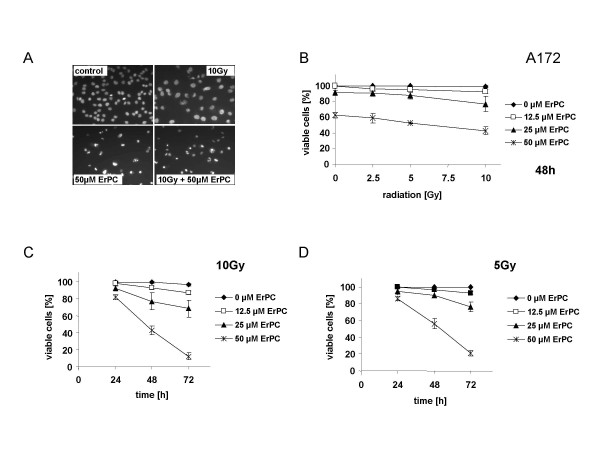
**ErPC increases cytotoxicity of ionizing radiation in A172 cells**. A172 cells were irradiated with a single dose of 0, 2.5, 5 or 10 Gy and subsequently treated with 0, 12.5, 25 or 50 μM ErPC as indicated. Induction of apoptosis and necrosis was quantified 24 h, 48 h and 72 h after treatment by fluorescence microscopy counting the cells with apoptotic and necrotic morphology upon combined staining with Hoechst33342 and PI. The percentage of viable cells was calculated as indicated in Fig.1. Data show **(A) **one representative of three independent experiments **(B, C, D) **or means ± s.d. ; n = 3. **(A) **Morphologic appearance of A172 cells upon treatment with medium (control), 10 Gy, 50 μM ErPC or 10 Gy and 50 μM ErPC. **(B) **Increased efficacy of ErPC in combination with ionizing radiation depends on the radiation dose and the ErPC-concentration. **(C **and **D) **Increased efficacy of ErPC in combination with 10 or 5 Gy depends on the treatment time.

Similar to the results obtained with T98G-cells, combined treatment with increasing concentrations of ErPC sensitized A172 cells to radiation-induced apoptosis (Fig. 4). As shown in Fig. 4A, irradiation with 10 Gy alone only induced growth arrest of A172 cells (decrease in cell density) without any morphological signs for induction of apoptosis. In contrast, treatment with 50 μM ErPC alone induced growth arrest and apoptosis of A172 cells. However, the level of apoptotic cells further increased by combined administration of both treatments (Fig. 4A). Increased cytotoxicity of the combination was dependent on drug-concentration and radiation dose (Fig 4B). While the combination of 12.5 and 25 μM ErPC only slightly increased the cytotoxic efficacy of ionizing radiation, the combination of 50 μM with ionizing radiation efficiently induced cell death yielding up to 57% cell kill at 50 μM ErPC combined with 10 Gy (Fig. 4B). Again, at all radiation doses tested the combined effect was clearly time- and concentration dependent with maximal cytotoxicity at 50 μM and 72 h of treatment (Fig. 4C+D and data not shown).

**Figure 5:**
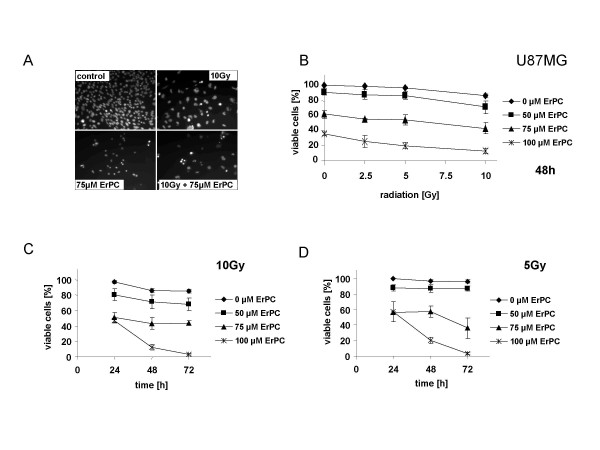
**Increased cytotoxicity of ionizing radiation in combination with ErPC in U87MG cells**. U87MG cells were irradiated with a single dose of 0, 2.5, 5 or 10 Gy and subsequently treated with 0, 50, 75 or 100 μM ErPC as indicated. Induction of cell death was quantified 24 h, 48 h and 72 h after treatment by fluorescence microscopy counting the cells with apoptotic and necrotic morphology upon combined staining with Hoechst 33342 and PI. The percentage of viable cells was calculated as indicated in Fig.1. Data show **(A) **one representative of three independent experiments or **(B, C, D) **means ± s.d.; n = 3. **(A) **Morphologic appearance of U87MG cells upon treatment with medium (control), 10 Gy, 75 μM ErPC or 10 Gy and 75 μM ErPC. **(B) **Concentration- and dose-dependent increase in cytotoxic efficacy of the combination. **(C **and **D) **Time-dependent increase in cytotoxicity of ErPC in combination with 10 or 5 Gy.

As mentioned above, 75 to 100 μM ErPC were required to induce significant growth arrest and apoptosis in U87MG cells (Fig. 1A, B, E). Therefore, to test putative sensitizing effects of ErPC on radiation-induced cell death in U87MG cells irradiation was combined with 0, 50, 75 and 100 μM ErPC. Photomicrographs of the cells treated for 48 h with 10 Gy, 75 μM ErPC or the combination reveal that irradiation alone yields small amounts of growth arrest and apoptosis while treatment with 75 μM ErPC induced strong growth arrest and increased amounts of apoptosis compared to radiation alone (Fig. 5A). However, combined treatment with 10 Gy and 75 μM ErPC resulted in a further rise in cell death-induction (Fig. 5A).

As shown in Fig. 5B, enhanced efficacy of the combination depended on the radiation dose and the ErPC-concentration (Fig. 5B). Similar to the results obtained with T98G and A172 cells, at all radiation doses tested the response of the combined treatment increased in a time- and concentration-dependent manner. However, in contrast to A172 and T98G cells, maximum induction of cell death was already observed 48 h after treatment (Fig. 5C+D and data not shown). Consistent with the comparably low sensitivity of U87MG cells to ErPC, massive rates of more than 80% cell kill required the presence of 100 μM ErPC (Fig. 5B–D).

### ErPC mediates additive to synergistic sensitization effects on radiation-induced apoptosis

**Figure 6:**
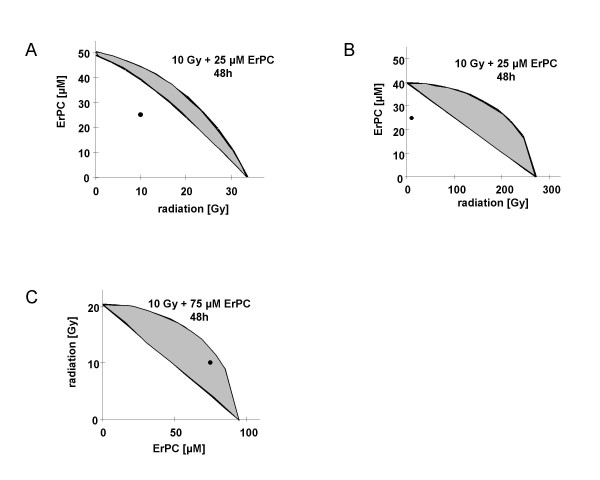
**ErPC sensitizes human malignant glioma cell lines to radiation-induced cell death**. Induction of apoptosis and clonogenic cell survival was evaluated in U87MG, A172 and T98G cells upon irradiation (1–10 Gy) or treatment with ErPC (0–100 μM). Cell death was quantified 24–72 h after treatment by fluorescence microscopy using combined staining with Hoechst 33342 and propidium iodide. The biomathematical evaluation of putative additive or synergistic effects of the combination was performed by isobologram analysis [52]. Analysis of combined treatment efficacy was performed with 10 Gy and 25 μM ErPC (T98G, A172), or 10 Gy and 75 μM ErPC (U87MG) 48 h after treatment. Values located within the envelope of additivity (grey region) are indicative for additive effects, values located below the envelope of additivity are indicative for synergistic increase in cytotoxicity. Combined treatment with ErPC increases cytotoxic efficacy of ionizing radiation (10 Gy, 48 h) **(A, B) **in a synergistic (T98G, A172 cells) or **(C) **additive manner (U87MG cells).

**Figure 7:**
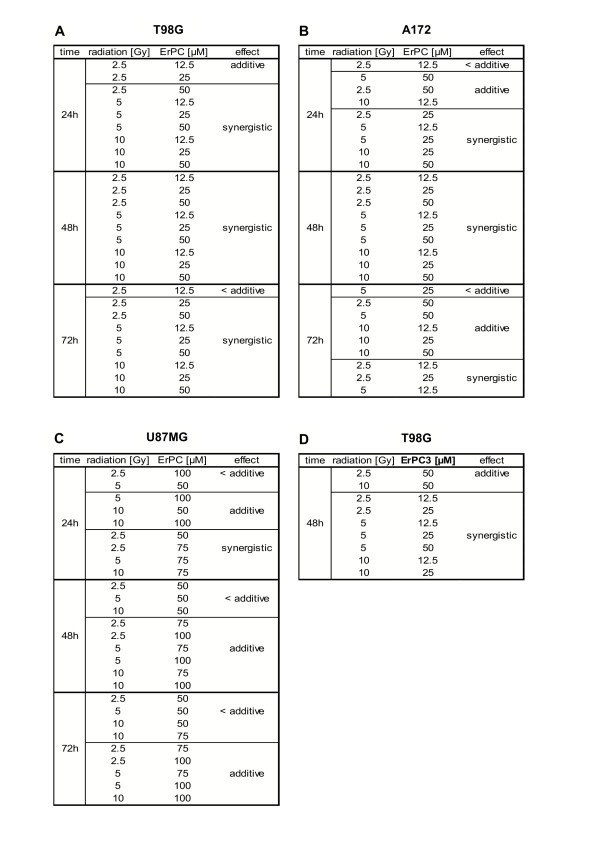
Results from isobologram analysis of combined treatment.

To determine how far the interactions between irradiation and ErPC-treatment in human malignant glioma cell lines were sub-additive, additive or even synergistic, biomathematical evaluation was performed by isobologram analysis. In general, sensitivity of malignant glioma cells depended on drug concentration, radiation dose and treatment time (Fig. 6+7). T98G were most responsive to combined treatment showing almost exclusively synergistic effects after 24 h, 48 h and 72 h of treatment. Combined treatment of A172 cells revealed sub-additive to synergistic effects after 24 h and 72 h, and synergistic effects after 48 h of treatment. U87MG were slightly less responsive compared to T98G and A172 with less than additive to synergistic effects at 24 h and sub-additive to additive effects at 48 and 72 h after treatment (Fig. 7A–C).

Representative analysis from selective combinations 48 h after treatment are represented in Fig. 6. In T98G and A172 cells a synergistic increase in cytotoxicity of the combination was observed after 48 h of treatment with 25 μM ErPC and 10 Gy (Fig. 6A+B), while in U87MG cells additive effects of 75 μM ErPC in combination with 10 Gy were found (Fig. 6C).

### ErPC3 sensitizes T98G cells to radiation-induced apoptosis

Based on the high responsiveness of T98G cells to ErPC alone and in combination with radiation therapy, we extended our studies on the ErPC-derivative ErPC3 (Erufosine™) which is more advanced in clinical development (Lars H. Lindner, unpublished data).

**Figure 8:**
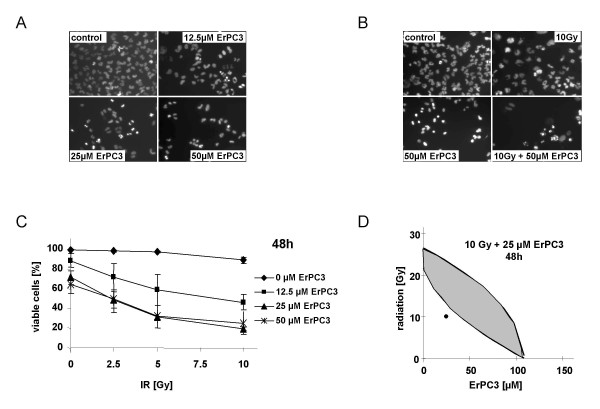
**ErPC3 exerts potent cytotoxic effects on T98G cells alone and in combination with ionizing radiation**. T98G were treated with 0–50 μM ErPC3 alone or in combination with 0, 2.5, 5 or 10 Gy for 48 h as indicated. Induction of apoptosis and necrosis was determined by fluorescence microscopy upon staining with Hoechst33342 and PI. The percentage of apoptotic and necrotic cells was then quantified by counting of the cells with apoptotic and necrotic morphology, respectively. The percentage of viable cells was calculated as indicated in Fig.1. Data show **(C) **means ± s.d., n = 3 or **(A, B, D) **one representative of three independent experiments. **(A) **ErPC3 induces growth arrest and apoptosis in a dose-dependent manner in T98G cells as indicated by a decrease in cell density and increase in cells with apoptotic morphology. **(B) **Combined treatment with ErPC3 sensitizes T98G cells to radiation-induced growth arrest and apoptosis. **(C) **Increased efficacy of ErPC3 in combination with ionizing radiation depends on the radiation dose and drug concentration, respectively. **(D) **Isobolgram analysis of combined treatment with 10 Gy and 25 μM ErPC3 after 48 h reveals synergistic effects.

In a first set of experiments cytotoxic efficacy of ErPC3 was evaluated in the most responsive T98G cells 48 h after treatment with the same drug concentrations as used for the ErPC-experiments (0, 12.5, 25 or 50 μM ErPC3). Similar to ErPC, its derivative ErPC3 turned out to be a potent inducer of growth arrest and apoptosis in T98G cells (Fig. 8A). In this regard, ErPC3 was already effective at concentrations of 12.5 μM and a more pronounced cytostatic and cytotoxic activity was observed at increased drug concentrations (Fig. 8A+C). Given the potent apoptosis inducing effects of ErPC3 we subsequently analyzed its putative sensitizing effects on radiation-induced cell death. As shown in Fig 8B combined treatment with ErPC3 and 10 Gy efficiently enhanced growth arrest and apoptotic cell death in T98G cells compared to either treatment alone as indicated by reduced cell density and enhanced numbers of cells with condensed chromatin and nuclear fragmentation, respectively. Increased efficacy of the combined treatment depended on the drug-concentration and the radiation-dose (Fig. 8C). Interestingly, maximal cytotoxicity of the combination with 81% cell death was already obtained with 25 μM ErPC3 in combination with 10 Gy (Fig. 8C). Evaluation of the interaction between ErPC3 and ionizing radiation by isobologram analysis revealed mostly synergistic effects as shown in Fig. 7D and 8D.

### Increased efficacy of the combined treatment is at least partially due to enhanced apoptosis levels

**Figure 9:**
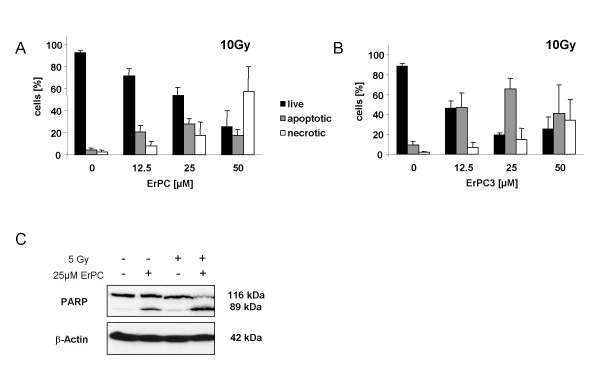
**Increased induction of apoptosis contributes to increased efficacy of the combination**. **(A, B) **T98G cells were treated with a single dose of 10 Gy and subsequently treated with 0, 12.5, 25 or 50 μM ErPC or ErPC3 as indicated. The amount of apoptotic and necrotic cells was determined 48 h after treatment by fluorescence microscopy upon staining with Hoechst33342 and PI and counting of the cells with apoptotic morphology and necrotic morphology, respectively. **(C) **T98G cells were treated with a single dose of 5 Gy and subsequently treated with 0 or 25 μM ErPC. Cleavage of the caspase-3 substrate PARP was determined by Western blotting of cytosolic extracts obtained 24 h after treatment. **(A) **Dose dependent increase in the percentage of apoptotic and necrotic T98G cells upon combined treatment with increasing concentrations of ErPC and 10 Gy. **(B) **Efficient Increase in the percentage of apoptotic T98G cells upon combined treatment with ErPC3 and 10 Gy. **(C) **Improved cleavage of PARP upon combined treatment with 25 μM ErPC and 5 Gy. Data show **(A, B) **means ± s.d., n = 3 or **(C) **one representative of three independent experiments.

In order to gain insight into the importance of apoptosis for synergistic cell death induction by combined treatment with ionizing radiation and ErPC or ErPC3 we first analyzed the prevailing mechanism of cell death upon combined treatment. As demonstrated in Fig. 9A+B, combined treatment with 10 Gy and various concentrations of ErPC or ErPC3 predominantely induced apoptosis compared to necrosis, with the exception of 50 μM ErPC in combination with 10 Gy. Interestingly, at equimolar drug concentrations ErPC3 sensitized T98G cells more efficiently to radiation-induced apoptosis than ErPC (Fig. 9A+B).

Specialized cellular proteases, the caspases have been identified as major executioners of apoptotic cell death. To further demonstrate the importance of apoptosis induction for the sensitizing effects on radiation-induced cell death we analyzed cleavage of the effector caspase-substrate PARP, a nuclear protein involved in DNA repair. While in control cells no PARP-cleavage could be detected, administration of 25 μM ErPC led to appearance of the cleaved PARP fragment (89 kDa), indicative for caspase-3 activation. In contrast, radiation up to 10 Gy was not sufficient to induce significant PARP-cleavage (Fig. 9C and data not shown). Enhanced cytotoxicity of combined treatment with 25 μM ErPC and 5 Gy was accompanied by a more prominent cleavage of PARP compared to ErPC-treatment alone, indicative for increased caspase-activation and apoptosis (Fig. 9C).

**Figure 10:**
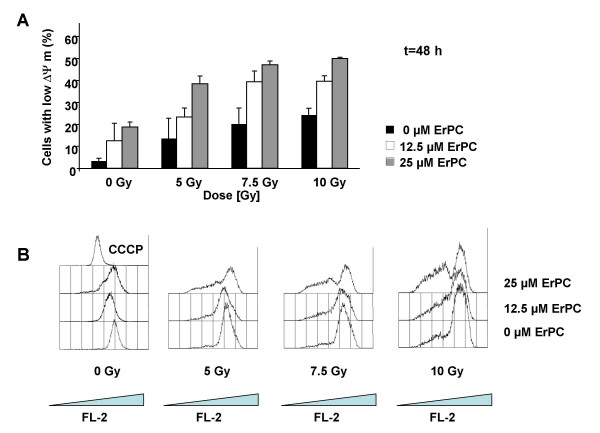
**Increased pro-apoptotic efficacy of the combination involves enhanced damage of the mitochondria**. T98G were irradiated with a single dose of 0, 2.5, 5 or 10 Gy and subsequently treated with 0, 12.5, 25 or 50 μM ErPC as indicated. Induction of apoptosis was quantified 48 h after treatment by FACS upon staining with TMRE. Data show **(A) **means ± s.d., n = 3 or **(B) **original histograms of one representative experiment.

Our earlier investigations revealed that apoptosis induction by ionizing radiation and ErPC involves alterations of mitochondrial function including breakdown of the mitochondrial membrane potential and release of cytochrome c. To quantify apoptosis induction by an additional standard method we analyzed therapy-induced breakdown of the mitochondrial membrane potential (Fig. 10). In agreement with the results obtained by quantification of cells with apoptotic nuclear morphology combined treatment with ErPC increased radiation-induced mitochondrial damage. These findings point to increased efficacy of the combination at the level of the mitochondria (Fig. 10A+B).

### ErPC and ErPC3 reduce colony formation ability of T98G cells and increase radiation-induced eradication of clonogenic T98G cells

**Figure 11:**
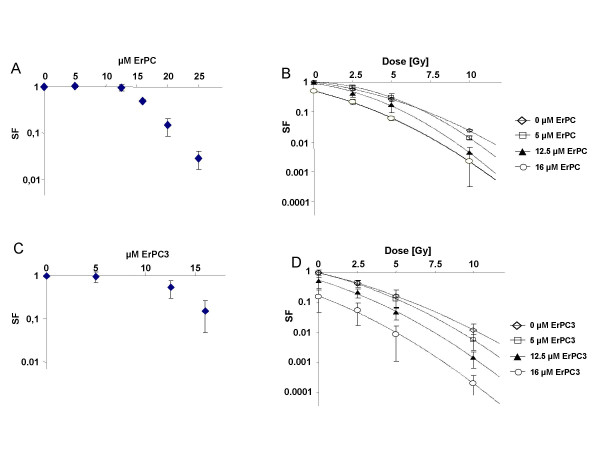
**ErPC and ErPC3 decrease the number of clonogenic T98G cells and increase radiation-induced eradication of clonogenic T98G cells**. Effects of ErPC and ErPC3 alone or in combination with ionizing radiation on clonogenic cell survival was determined by standard colony formation assays. 24 h after plating T98G cells were treated with increasing concentrations of **(A) **ErPC or **(C) **ErPC3 alone or **(B, D) **in combination with ionizing radiation with 0, 2.5, 5 and 10 Gy. In the latter case, ErPC or ErPC3 were added 10 min after irradiation. Subsequently, cells were incubated under standard culturing conditions up to 4 weeks. For determination of colony formation cells were fixed in 3.7% paraformaldehyde and 70% ethanol and stained with 0.05% Coomassie blue. Colonies composed of at least 50 cells were counted. Data represent means ± s.d., n = 3

Up to now our data indicated that ErPC and ErPC3 increase sensitivity of AC/GBM cell lines to radiation induced cell death, in particular apoptosis. To gain more insight into cytotoxic efficacy of ErPC/ErPC3 treatment alone and in combination with radiation, standard colony formation assays were performed as a clinical relevant endpoint. As shown in Figure 11A+C ErPC and ErPC3 reduced clonogenic survival of T98G at concentrations of more than 12.5 μM. A prominent reduction of the surviving fraction was obtained upon treatment with 25 μM ErPC. ErPC3 even more efficiently reduced clonogenic cell survival of T98G cells: While 16 μM ErPC3 were sufficient to eradicate 90% of clonogenic tumor cells, 20 μM ErPC were required to induce the same effect (Fig. 11A+C).

In a next set of experiments we then tested whether combined treatment with ErPC or ErPC3 would alter eradication of clonogenic tumor cells in response to ionizing radiation. Despite the above mentioned resistance of T98G cells to radiation-induced apoptosis irradiation was able to reduce clonogenic cell survival in a dose-dependent manner (Fig. 11B+D). However, the combination with ErPC or ErPC3 led to a further decrease in the survival of clonogenic T98G cells upon irradiaton (Fig. 11B+D). As visualized in Fig. 11B and 11D, combined treatment of irradiated cells with increasing concentrations of ErPC and ErPC3 led to a parallel shift of the response curves at least at the low dose range indicative for additive effects, while at higher doses additivity was not reached. Interestingly, combined treatment with 16 μM ErPC3 and ionizing radiation was more efficient in eradication of clonogenic tumor cells than the respective combination with equimolar ErPC-concentrations.

## Discussion

Based on the hypothesis that synthetic phospholipid derivatives and ionizing radiation induce apoptosis via distinct primary targets to trigger the intrinsic death pathway, cytotoxic efficacy of combined treatment with both therapies was evaluated in human malignant glioma cell lines *in vitro*. In our investigation we demonstrate for the first time that the prototypical intravenously applicable APC-derivatives ErPC and ErPC3 increase the radiation response of human malignant glioma cell lines. In short term assays ErPC and ErPC3 enhanced sensitivity of these highly resistant cells to radiation-induced cell death, including apoptosis. Any combination of radiation with ErPC was more effective than either treatment alone; depending on the cell type, treatment time, dose level and drug-concentration sub-additive, additive or synergistic effects were observed. In long term colony formation assays ErPC and ErPC3 were shown to efficiently kill clonogenic tumor cells on their own and to increase radiation-induced eradication of clonogenic tumor cells upon combined treatment in an additive manner.

The observation of potent short term cytostatic and cytotoxic effects of ErPC and ErPC3 on human malignant glioma cell lines *in vitro *is consistent with earlier investigations in diverse human cancer cell lines including malignant glioma ([24,29,30] and unpublished data). As a more clinically relevant end point we demonstrate that these membrane targeted drugs reduce colony formation in long term assays, indicative for eradication of clonogenic tumor cells.

Up to now, only a few *in vitro *studies tested the efficacy of synthetic phospholipid analogs in combination with ionizing radiation. In this regard, the APC-derivatives hexadecylphosphocholine (HePC) and Octadecyl-(N, N-dimethyl-piperidinio-4-yl)-phosphate (D-21266; Perifosine™) enhanced radiation-induced apoptosis of human leukemic cell lines in an additive, the prototypical alkyllysophospholipid Edelfosine (Et-18-OCH_3_; 1-*O*-Octadecyl-2-*O*-methyl-*rac*-glycero-3-phosphocholine) even in a synergistic manner. Similar to ErPC and ErPC3, HePC was able to overcome resistance of human tumor cells to radiation-induced apoptosis. In line with our observations, combined treatment of human epithelial tumor cell lines with Et-18-OCH_3 _or HePC and ionizing radiation also enhanced eradication of clonogenic tumor cells in colony formation assays [22]. Thus, our data are consistent with these reports on profitable *in vitro *effects of combined treatment with synthetic phospholipid derivatives and ionizing radiation regarding radiation-induced apoptosis and clonogenic cell kill in human cancer cell lines.

Interestingly, mechanistic investigations point to a critical role of apoptosis induction for enhanced efficacy of the combination. In this regard, notably for ErPC3 induction of apoptosis was the major mode of cell death after short time treatment. On molecular level, synergistic effects on apoptosis induction upon combined treatment were reflected by increased cleavage of the caspase-3 substrate PARP. Moreover, synergistic effects on apoptosis induction involved enhanced mitochondrial damage. Thus, beneficial effects of these novel drugs on efficacy of ionizing radiation may at least partially be due to increased apoptosis induction at the level of the mitochondria. However, the significance of apoptosis for long term radiation responses of tumor cells and normal tissues is still controversial and was suggested to depend on the cellular system [34,35]. In our hands quantification of apoptosis induction in short term assays was not predictive for the radiation response in colony formation assays with respect to ionizing radiation alone. However, efficient induction of apoptosis upon treatment with ErPC alone or in combination with ionizing radiation was associated with increased eradication of clonogenic tumor cells in colony formation assays. Thus, the combination of ionizing radiation and the apoptosis modulators ErPC and ErPC3 can clearly increase efficacy of radiation treatment. Similar results were recently obtained using ionizing radiation in combination with the proapoptotic tumor necrosis factor alpha related apoptosis inducing ligand TRAIL [36,37] and the protein kinase inhibitor PKC412, respectively [38]. However, it has to be considered, that cell cycle arrest in G2 also contributes to the toxicity of ErPC/ErPC3 in human glioblastoma cells (data not shown).

Although several synthetic phospholipid derivatives displayed promising antineoplastic activity in preclinical investigations, it has to be taken into account that only few drugs may be suited for future clinical drug development. Despite its potent antineoplastic action *in vitro *the *in vivo *activity of the prototypical alkyllysophospholipid derivative Et-18-OCH_3 _is only moderate. This was attributed to the high level of biotransformation after systemic application that results in a lack of tissue accumulation [39]. Hemolytic side effects of HePC, the first APC-derivative successfully introduced into the clinic, preclude its intravenous application. Consequently, clinical use of HePC is either limited to topical application as an effective palliative treatment option for skin metastases of breast cancer patients as well as for cutaneous malignant lymphoma (Miltex™) [40-44] or to oral application (Impavido™). Daily doses of 100 mg HePC which have been shown to be insufficient for cancer treatment have been proven to cure visceral leishmaniasis [45]. Dose escalation of orally given HePC is prevented by gastrointestinal toxicity.

Perifosine, a heterocyclic APC, showed promising antineoplastic activity in preclinical investigations [46] and already entered clinical Phase I and Phase II trials to test feasibility and tolerability of oral administration of the drug alone and in combination with radiotherapy in patients with advanced solid tumors. Oral administration of Perifosine is safe and mainly results in fatigue and gastrointestinal side-effects while no hematological toxicity could be observed [19,47-50]. Unfortunately, no significant clinical activity was found after single drug administration in patients with metastatic melanoma and androgen independent prostate cancer [49,50].

In contrast to the above mentioned drugs, ErPC and its derivative ErPC3 lack hemolytic side effects and thus constitute the first synthetic phospholipid analogs that are suited for intravenous administration. After repeated i.v. applications of nontoxic drug doses ErPC accumulates in diverse tissues of healthy rats including the brain tissue [31]. Fortunately, ErPC and ErPC3 are even more potent than HePC in preclinical investigations [29,51].

Intriguingly, in our *in vitro *study ErPC3 was a more effective inducer of apoptosis in T98G cells than ErPC when given alone and in combination with ionizing radiation. Moreover, in colony formation assays ErPC3 also proved to be the more active when used as single drug or in combination with ionizing radiation. In this regard, similar eradication of clonogenic tumor cells required 16 μM ErPC3 or 20 μM ErPC, respectively. The same holds true for combined treatment with 10 Gy and 16 μM ErPC3 compared to 10 Gy and 25 μM ErPC.

The above mentioned findings on improved antineoplastic activity compared to ErPC together with its higher solubility in aqueous solutions that allows simplified intravenous administration *in vivo*, favor ErPC3 for further clinical development. Consequently, a clinical Phase I trial was initiated at the Department of Internal Medicine III, University Hospital Grosshadern, Munich, Germany, to test feasibility and tolerability of intravenous administration of ErPC3 to patients with advanced malignancies. As the maximum tolerated dose (MTD) of ErPC3 has not yet been reached, patient recruitment goes on (Dr. L. H. Lindner, Dept. of Internal Medicine III, University Hospital Grosshadern, Munich, Germany, personal communication).

In summary our study demonstrates increased efficacy of ionizing radiation in combination with the proapoptotic membrane targeted apoptosis modulators ErPC and ErPC3 in human malignant glioma cell lines *in vitro*. Both drugs sensitized human malignant glioma cell lines to radiation-induced cell death including apoptosis and enhanced radiation-induced eradication of clonogenic tumor cells. The improved efficacy of ErPC3 compared to ErPC make this APC derivative a promising tool for innovative combined treatment approaches in the therapy of patients suffering from malignant glioma. The molecular requirements for the increased efficacy of radiation therapy in combination with ErPC and ErPC3 require further definition.

## Methods

### Chemicals and drugs

ErPC and ErPC3 were synthesized by H. Eibl, Max Planck Institute of Biophysical Chemistry, Goettingen, Germany. For in vitro experiments, ErPC was dissolved in 200 μl ethanol, and diluted with RPMI1640 medium supplemented with 10% (v/v) fetal calf serum to a concentration of 10 mM (stock solution). The final ethanol concentrations in the tissue culture experiments were below 0.05% (v/v). ErPC3 was dissolved in RPMI1640 medium supplemented with 10% (v/v) fetal calf serum to a concentration of 10 mM (stock solution) without prior dissolution in ethanol. Hoechst33342 (Calbiochem, Bad Soden, Germany) was dissolved in distilled water as a 1.5 mM stock solution. Propidium Iodide (Sigma) was dissolved in distilled water as a 5 mg/ml stock solution.

Rabbit anti-full length anti-PARP and rabbit anti-cleaved PARP were from Cell Signaling (New England Biolabs, Schwalbach/Taunus, Germany). Mouse β-actin was from Sigma. HRP-conjugated anti-rabbit secondary antibodies were obtained from Amersham-Biosciences, Freiburg, Germany.

All other chemicals were purchased from Sigma-Aldrich (Deisenhofen, Germany) if not otherwise specified.

### Cell lines, cell culture and cellular treatment

T98G, U87MG and A172 astrocytoma/glioblastoma cell lines were from ATCC (Bethesda, Maryland, USA). For all experiments cells were grown in RPMI 1640 medium supplemented with 10% (v/v) fetal calf serum (Gibco Life Technologies, Eggenstein, Germany) and maintained in a humidified incubator at 37°C and 5% CO_2_.

Irradiation was performed at room temperature with 6 MV photons from Siemens or Elekta linear accelerators with a dose rate of 2 or 4 Gy per min, respectively.

### Determination of apoptosis

Cell death was analyzed by fluorescence microscopy upon combined staining of the cells with Hoechst33342 and propidium iodide (PI) to discriminate between apoptotic and necrotic cells. In brief, cells were incubated with Hoechst33342 at a final concentration of 1.5 μM and PI at a final concentration of 2.5 μg/ml for 10 min. Cell morphology was determined by fluorescence microscopy (Zeiss Axiovert 200, Carl Zeiss, Jena, Germany) using a G365/FT395/LP420 filterset. Cells were analyzed at x40 magnification and documented using a CCD camera device (Zeiss Axiocam MR). Apoptotic cells (blue or rose stained nuclei with apoptotic nuclear morphology) and necrotic cells (rose stained nuclei without fragmentation) were quantified by cell counting.

### Determination of PARP-cleavage

PARP cleavage was determined by Western blot analysis of cytosolic extracts. To this end, cells (1 × 10^7^/ml) were lyzed for 10 min at 99°C in CST lysis buffer (62.5 mM Tris-HCl (pH 6,8), 2% (w/v) SDS, 10% (v/v) glycerol, 50 mM DTT, 0.01% (w/v) bromphenolblue). 20 μg lysate were separated by SDS-PAGE and blotted onto PVDF-membranes (Roth, Karlsruhe, Germany). Blots were blocked for 1 h in PBS buffer containing 0.05% (v/v) Tween 20 and 5% (w/v) non fat dried milk. The membrane was incubated over night at 4°C with the respective primary antibody (anti-PARP; anti-cleaved PARP; 1:1000). After repeated washings with TBS/Tween-20 (0.05%, v/v) the membrane was incubated for 1 h at room temperature with the secondary antibody (anti-IgG-AP 1:2000, Santa-Cruz-Biotech, Heidelberg, Germany) and again washed several times with TBS/Tween. The detection of antibody binding was performed by enhanced chemoluminescence staining (ECL Western blotting analysis system, Amersham-Biosciences, Freiburg, Germany). Equal protein loading was confirmed by Coomassie stain and β-actin detection.

### Determination of mitochondrial membrane potential

The mitochondrial transmembrane potential (ΔΨm) was analyzed by flow cytometry using the ΔΨm-specific stain TMRE (tetramethylrhodamine-ethylester-perchlorate) (Molecular Probes, Mobitech, Goettingen Germany). To this end, cells were loaded for 30 min at 37°C with 25 nM TMRE and subsequently analyzed by flow cytometry. Preincubation with 1 μM of the proton ionophore CCCP (carbonylcyanide-m-chlorophenylhydrazone) was used as a positive control for complete depolarization.

### Colony formation assays

Exponentially grown cells were harvested and seeded in 6 well tissue culture plates or flasks at a density of 50 to 128 000 cells depending on irradiation and ErPC doses. After 24 h cells were irradiated (2.5, 5 or 10 Gy), treated with ErPC (12.5–100 μM) or submitted to combined treatment. Subsequently, cells were incubated under standard culturing conditions (37°C/5% CO_2_) up to 4 weeks depending on treatment. For determination of colony formation cells were fixed in 3.7% formaldehyde and 70% ethanol. Fixed cells were stained with 0.05% Coomassie blue.

Colonies of at least 50 cells were counted. The surviving fraction of treated cultures was calculated by dividing the number of colonies by the plating efficiency of untreated cells. The survival curve was established by plotting the log of the surviving fraction versus the irradiation dose.

### Statistical analysis

Efficacy of the combined treatment modalities was evaluated by isobologram analysis [52]. Based on the measured values for single treatment regimens, data for combined treatment were extrapolated defining a calculated area of additive treatment response (envelope of additivity) [52]. The given results of the experiments were then compared to the calculated data: values within the envelope of additivity are representative for an additive effect, while values below this envelope indicate a synergism between both treatment modalities. In contrast, values above the envelope of additivity are indicative for sub-additive effects.

## Abbreviations

AC, astrocytoma; APC, alkylphosphocholine; ErPC, erucylphosphocholine; ErPC3, Erucylphosphohomocholine, Erufosine™; ET18-OCH_3_, Edelfosine; GBM, glioblastoma; HePC, Hexadecylphosphocholine

## Competing interests

The author(s) declare that they have no competing interests.

## Authors' contributions

AR contributed significantly to data acquisition, data analysis and drafting the manuscript. RH contributed significantly to data acquisition, data analysis and drafting the manuscript. LHL and MS performed critical revision of the manuscript. HE synthesized and provided ErPC and ErPC3 for the analysis. WB significantly contributed to data analysis. CB participated in the conception of the study and interpretation of data. VJ performed conception and design of the study and substantially contributed to interpretation of data, drafting of the manuscript, critical revision of the manuscript and final approval. All authors read and approved the final manuscript.
